# Editorial

**Published:** 1856-12

**Authors:** 


### THE MEDICAL EXAMINER.

### PHILADELPHIA, DECEMBER, 1856.

With the present number our Editorial connection with the Examiner ceases. Having expressed our determination several months since to this effect to its worthy publishers, an arrangement was effected by them with Drs. S. D. Gross and T. G. Richardson, which, we are confident, will give general satisfaction. By this arrangement the Medical Examiner and the Louisville Review have been combined, and will constitute a new Journal of one hundred and sixty pages, under the title of the North American Medico-Chirurgical Review, to be issued every alternate month. The subscribers to both Journals will thus receive a far larger amount of matter than either previously afforded them, while the well-known characters and experience of the gentlemen who take charge of the Review, are a guarantee that it will be conducted in an honorable and independent spirit, and with a sole aim to the interests of their profession.

In thus terminating our duties as Editor, we beg to express to our collaborators and other professional friends our grateful acknowledgments for their kind and valuable assistance. To them and to our readers, with whom we have held pleasant intercourse during the last three years, we tender a friendly and respectful *farewell.*

Health of Philadelphia.—Scarlet Fever has been very prevalent and fatal in Philadelphia during the present year. During the 1st quarter, beginning with Dec. 29th, 1855, the deaths from it were 189; during the 2d, 186; and during the 3d, ending Sept. 27, 179. Since that period, the weekly mortality from it has been 17, 17, 14, 22, 22, 35, 34, 27. During the last week, ending Nov. 29th, it rose to the unprecedented number of 45. Should there be no abatement of the disease during the remaining four weeks of the year, ending Dec. 27th, the deaths from it during the whole year would amount to 967.

On referring to Dr. Jewell's Tables in our February No., we find the deaths from scarlet fever during the last eight years, beginning with 1848, were 172, 242, 439, 400, 433, 391, 165 and 163. It will thus be seen that the mortality from it during the last five weeks, exactly equals that of the whole preceding year.

Copland's Medical Dictionary.—We learn authoritatively, that the distinguished author of this invaluable work is engaged in preparing an Index, so that the concluding portion will ere long be before the profession.

Abstract of Meteorological Observations for October, 1856, made in the Twenty-fourth Ward, Philadelphia. By Prof. James A. Kirkpatrick.

| 1856. October. | Barometer. Reduced to 32°. Daily Mean | Barometer. Mean Daily Range. | Thermometer. Daily Mean | Thermometer. Mean Daily Range | Thermometer. Extreme Daily Range | Solar Radiation. 2 P.M. | Rel. Humid. 2 P.M. | Force of Vapor 2 P.M. | Dew Point 2 P.M. | Rain | Prevailing Winds. | Remarks. |
|---|---|---|---|---|---|---|---|---|---|---|---|---|
| | Inches. | Inches. | Deg. | Deg. | Deg. | Deg. | Hunds. | Inches. | Deg. | Inches. | Points. | |
| 1 | 29.530 | .196 | 47.7 | 15.3 | 15 | | 37 | .156 | 23.4 | | W. | Morning and afternoon cloudy; evening clear. |
| 2 | 29.730 | .199 | 49.0 | 8.0 | 22 | | 29 | .130 | 24.2 | | SSW. | Morning and evening clear, afternoon cloudy; *ice* in exposed situations; *heavy hoar frost.* |
| 3 | 29.747 | .033 | 54.8 | 7.5 | 20 | 16 | 38 | .228 | 37.9 | | (Var.) | Clear. |
| 4 | 29.904 | .157 | 59.0 | 5.5 | 26 | 15 | 48 | .353 | 49.4 | | SW. | Clear. |
| 5 | 29.892 | .034 | 65.8 | 6.8 | 24 | 18 | 57 | .527 | 60.5 | | S. | Morning and afternoon cloudy, evening clear. |
| 6 | 29.796 | .096 | 69.0 | 3.2 | 19 | 21 | 69 | .638 | 66.0 | | SW. | Morning cloudy; afternoon and evening clear. |
| ☽ 7 | 29.931 | .135 | 58.0 | 11.0 | 13 | 9 | 48 | .300 | 45.1 | | NW. | Morning cloudy; afternoon and evening clear. |
| 8 | 30.005 | .074 | 56.0 | 2.0 | 19 | 15 | 53 | .329 | 47.5 | | NW. | Clear. |
| 9 | 29.961 | .044 | 59.8 | 3.8 | 26 | 14 | 54 | .422 | 54.3 | | SW. | Clear. |
| 10 | 29.847 | .114 | 63.3 | 3.5 | 21 | | 60 | .518 | 60.0 | | WSW. | Clear. |
| 11 | 29.810 | .036 | 66.0 | 2.7 | 26 | 24 | 50 | .477 | 57.7 | | S. | Clear. *Thermometer highest* 78°. |
| 12 | 29.836 | .026 | 64.2 | 4.8 | 19 | 25 | 56 | .483 | 58.0 | | (Var.) | Morning cloudy; aft. and evening clear. |
| ○ 13 | 29.594 | .242 | 65.8 | 4.8 | 19 | 15 | 63 | .510 | 59.6 | | (Var.) | Cloudy. |
| 14 | 29.763 | .188 | 42.3 | 23.5 | 3 | | 51 | .142 | 26.2 | 0.285 | N. | Cloudy; morning *rain.* |
| 15 | 30.011 | .248 | 39.2 | 4.5 | 10 | 5 | 38 | .115 | 21.4 | | N. | Morning and aft. cloudy; evening clear. |
| 16 | 29.952 | .072 | 43.7 | 6.8 | 13 | 7 | 39 | .167 | 30.1 | | N. | Clear. *Barometer highest* 30.025. |
| 17 | 29.703 | .249 | 51.0 | 8.0 | 12 | | 87 | .362 | 50.1 | 0.906 | NE. | Cloudy; afternoon and evening *rain.* |
| 18 | 29.732 | .143 | 52.0 | 3.7 | 8 | 5 | 63 | .295 | 44.6 | | (Var.) | Morning and afternoon cloudy; evening clear. |
| 19 | 29.881 | .149 | 54.0 | 3.0 | 16½ | 8½ | 58 | .318 | 46.6 | | W. | Morning and afternoon cloudy: evening clear. |
| ☾ 20 | 29.947 | .066 | 56.0 | 2.0 | 17 | 8 | 48 | .300 | 45.1 | | NW. | Morning and afternoon cloudy; evening clear. |
| 21 | 29.928 | .035 | 60.0 | 4.0 | 22 | 22 | 44 | .322 | 46.9 | | (Var.) | Morning and evening clear; afternoon cloudy. |
| 22 | 29.642 | .286 | 63.3 | 3.3 | 25 | 4 | 73 | .433 | 55.0 | 0.031 | (Var.) | Morning and evening clear; afternoon cloudy; night *rain.* |
| 23 | 29.591 | .130 | 57.7 | 11.0 | 15 | | 68 | .419 | 54.1 | | NW. | Cloudy. |
| 24 | 29.867 | .276 | 36.7 | 21.0 | 11 | 6 | 26 | .071 | 10.9 | | NW. | Clear. |
| 25 | 29.891 | .066 | 40.8 | 5.8 | 21 | 6 | 44 | .152 | 27.8 | | SW. | M. clear; aft. and ev. cloudy. *Thermometer lowest* 28°. |
| 26 | 29.798 | .094 | 49.5 | 8.7 | 15½ | 5½ | 70 | .343 | 48.7 | | W. | Morning and afternoon cloudy; evening clear. |
| 27 | 29.674 | .124 | 53.0 | 6.5 | 11 | 2 | 74 | .308 | 45.7 | 0.029 | E. | Cloudy; *rain* during the night. |
| ● 28 | 29.553 | .123 | 47.0 | 6.7 | 12 | 5 | 39 | .167 | 30.1 | | W. | Clear. |
| 29 | 29.687 | .134 | 42.7 | 4.3 | 16 | 9 | 34 | .125 | 23.3 | | SW. | Clear. |
| 30 | 29.466 | .221 | 51.7 | 9.0 | 23 | 5 | 42 | .242 | 39.4 | 0.052 | (Var.) | Cloudy; evening *rain.* *Bar. lowest* 29.373. |
| 31 | 29.565 | .152 | 37.0 | 14.7 | 8 | 8 | 36 | .088 | 15.6 | | W. | M. and aft. cloudy; ev. clear. *Snow* in the afternoon, flakes large but few. |
| Means for Oct. 1856 | 29.782 | .133 | 53.4 | 7.3 | 17 | 11 | 52 | .305 | 42.3 | 1.303 | N. 87° 45′ W. 34-100. | |

